# Carbon Nanotube versus Graphene Nanoribbon: Impact of Nanofiller Geometry on Electromagnetic Interference Shielding of Polyvinylidene Fluoride Nanocomposites

**DOI:** 10.3390/polym11061064

**Published:** 2019-06-20

**Authors:** Mohammad Arjmand, Soheil Sadeghi, Ivonne Otero Navas, Yalda Zamani Keteklahijani, Sara Dordanihaghighi, Uttandaraman Sundararaj

**Affiliations:** 1 School of Engineering, University of British Columbia, Kelowna, BC V1V 1V7, Canada; s.dordanihaghighi@ubc.ca; 2 Department of Chemical and Petroleum Engineering, University of Calgary, Calgary, AB T2N 1N4, Canada; soheilsadeghi64@gmail.com (S.S.); ivonne.253@gmail.com (I.O.N.); yalda.zamaniketeklah@ucalgary.ca (Y.Z.K.)

**Keywords:** carbon nanotube, graphene nanoribbon, electromagnetic interference shielding, electrical conductivity, molecular simulation, rheology

## Abstract

The similar molecular structure but different geometries of the carbon nanotube (CNT) and graphene nanoribbon (GNR) create a genuine opportunity to assess the impact of nanofiller geometry (tube vs. ribbon) on the electromagnetic interference (EMI) shielding of polymer nanocomposites. In this regard, GNR and its parent CNT were melt mixed with a polyvinylidene fluoride (PVDF) matrix using a miniature melt mixer at various nanofiller loadings, i.e., 0.3, 0.5, 1.0 and 2.0 wt%, and then compression molded. Molecular simulations showed that CNT would have a better interaction with the PVDF matrix in any configuration. Rheological results validated that CNTs feature a far stronger network (mechanical interlocking) than GNRs. Despite lower powder conductivity and a comparable dispersion state, it was interestingly observed that CNT nanocomposites indicated a highly superior electrical conductivity and EMI shielding at higher nanofiller loadings. For instance, at 2.0 wt%, CNT/PVDF nanocomposites showed an electrical conductivity of 0.77 S·m^−1^ and an EMI shielding effectiveness of 11.60 dB, which are eight orders of magnitude and twofold higher than their GNR counterparts, respectively. This observation was attributed to their superior conductive network formation and the interlocking ability of the tubular nanostructure to the ribbon-like nanostructure, verified by molecular simulations and rheological assays.

## 1. Introduction

In recent years, major concerns have been raised over electromagnetic pollution due to the widespread use of electronics and telecommunication systems [1]. Polymers have gained popularity for electromagnetic interference (EMI) shielding applications because they are lightweight, resistant to corrosion, flexible, and have a lower cost than metals. As pristine polymers are insulative and thus transparent to electromagnetic waves, the incorporation of conductive fillers into the polymers is known as a robust technique to make polymers sensitive to electromagnetic waves [2,3,4].

Carbonaceous nanomaterials, including carbon nanotubes (CNTs), graphene nanoplatelets (GNPs) and carbon fiber, hold high esteem as conductive nanofillers due to their high aspect ratio, unique graphitic structure, high electrical conductivity, large surface area, and low synthesis cost [5,6,7]. So far, many theoretical and experimental studies have been devoted to developing conductive filler/polymer nanocomposites (CPNs) containing CNTs and GNPs for EMI shielding applications [1]. It is known that the EMI shielding of CPNs is a strong function of fillers’ intrinsic characteristics, such as electrical conductivity, dielectric properties, physical and geometrical features, and so forth [8]. CNTs and GNPs are considered as one-dimensional and two-dimensional lattice structures, respectively, featuring *sp*^2^ hybridized carbon atoms. Although both showcase truly excellent charge transfer and electronic properties, making them superb to improve the electrical conductivity of CPNs, they keep remarkably distinct geometrical features.

Comparison of the impact of different geometries of CNTs (tubular) and GNPs (planar) on electrical conductivity and EMI shielding of CPNs has been the focus of many theoretical and experimental studies. According to a theoretical study by Xie et al. [9], CPNs containing GNPs show higher electrical conductivity than those with CNTs, and as such they are interpreted as having a better conductive network. To the contrary, using Monte Carlo simulation, Safdari et al. [10] suggested an enhanced conductive network formation for CNTs compared to GNPs in an epoxy matrix. Martin-Gallego et al. [11] studied the rheological and electrical percolation of CNTs and GNPs in epoxy nanocomposites and claimed a stronger network in the CNT system, thus rendering CPNs with higher viscosity and electrical conductivity. Du et al. [12] compared the electrical properties of CNTs and GNPs in polyethylene nanocomposites and reported a much lower electrical percolation for the CNT systems. They claimed that CNTs created a three-dimensional conductive network in the polymer matrix, while GNPs ended up with a two-dimensional conductive structure. They ascribed this difference to the easier aggregation of GNPs, due to folding, rolling, and minor interlocking. 

Graphene nanoribbons (GNRs), narrow stripes of graphene, are fabricated by unzipping CNTs [13]. Identical to their parent CNTs, GNRs possess exceptional electronic and charge-transfer properties while featuring an ultra-high aspect ratio, frequent edge sides, and a more effective surface area than CNTs [14]. These properties suggest they would make a promising conductive nanofiller. Furthermore, under strictly controlled unzipping conditions, GNRs might contain only a few layers of ribbon, easing their exfoliation in the host polymer compared to GNPs [15]. Despite the presence of a lot of studies on CNTs and GNPs towards CPNs with enhanced physical properties, there are very few studies on GNR/polymer nanocomposites [16,17,18]. Furthermore, GNR is an exciting material as it has a geometry (ribbon-like) between CNT (tubular) and GNP (planar).

It is well known that EMI shielding is a strong function of the conductive network, that is, at a fixed nanofiller loading, a CPN with a better conductive network indicates superior EMI shielding [19,20]. In this regard, the similar molecular structures but distinct geometries of GNR and its parent CNT create a genuine opportunity to investigate the effect of nanofiller geometry (tube vs. ribbon) on conductive network formation and thus EMI shielding. To the best of our knowledge, the literature contains no research study comparing the EMI shielding of CPNs containing CNT and GNR. To strengthen our proceeding, besides the EMI shielding measurement, we employed molecular simulations, rheology, and electrical conductivity as direct or indirect evidence of conductive network formation in the developed nanocomposites. 

## 2. Experimental

### 2.1. Materials

Parent CNTs and GNRs were donated by AZ Electronic Materials, Branchburg, NJ, USA. According to the manufacturer, the GNRs were synthesized from the parent multi-walled CNTs using an Na/K alloy intercalation. In this technique, multi-walled CNTs are treated with Na/K alloy in 1,2-dimethoxyethane (DME) for several days, leading to intercalation of Na/K between the walls of CNTs. The intercalation leads to partial longitudinal cracking of the walls as they swell. Further details regarding this procedure are provided elsewhere [13]. Figure S1 depicts TEM images of parent CNT and GNR, and schematics of longitudinally fully unzipped and partially unzipped CNT [17]. It is worth noting that thermogravimetric analysis and Raman spectroscopy revealed inferior crystallinity and an abundance of edge atoms in GNR compared to CNT [16].

Poly(vinylidene fluoride) (PVDF), as the polymer matrix, was purchased from 3M Canada (Grade: 11008/0001) with an average density of 1.78 g/cm^3^ and a melting point of 160 °C. PVDF is a favorable polymer matrix for EMI shielding, by virtue of its combination of flexibility, low weight, low thermal conductivity, and high chemical corrosion and heat resistance. Moreover, PVDF features polar crystalline structures (β-phase), contributing to enhanced interaction with conductive nanofillers [21,22,23].

The nanofillers were melt-mixed with the PVDF matrix using the Alberta Polymer Asymmetric Minimixer (APAM) at 240 °C and 235 rpm. APAM has one-rotor configuration featuring a mixing cup, enclosed by a heating band. The cup has an inner diameter of 13 mm and a height of 25 mm. The rotor is similar to a roller blade [24]. Nanocomposites were developed at a broad spectrum of nanofiller contents, i.e., 0.3, 0.5, 1.0, and 2.0 wt%. Samples were compression molded into a circular shape (0.5 mm thickness) for rheological characterizations, and into a rectangular shape (1.1 mm thickness) for electrical and EMI shielding characterizations. Compression molding was performed with a Carver compression molder (Carver Inc., Wabash, IN, USA) at 240 °C under 38 MPa pressure for 10 min. Further information regarding the materials and nanocomposites preparation can be found in our previous study [17]. 

### 2.2. Molecular Simulations

**Figure 1:**
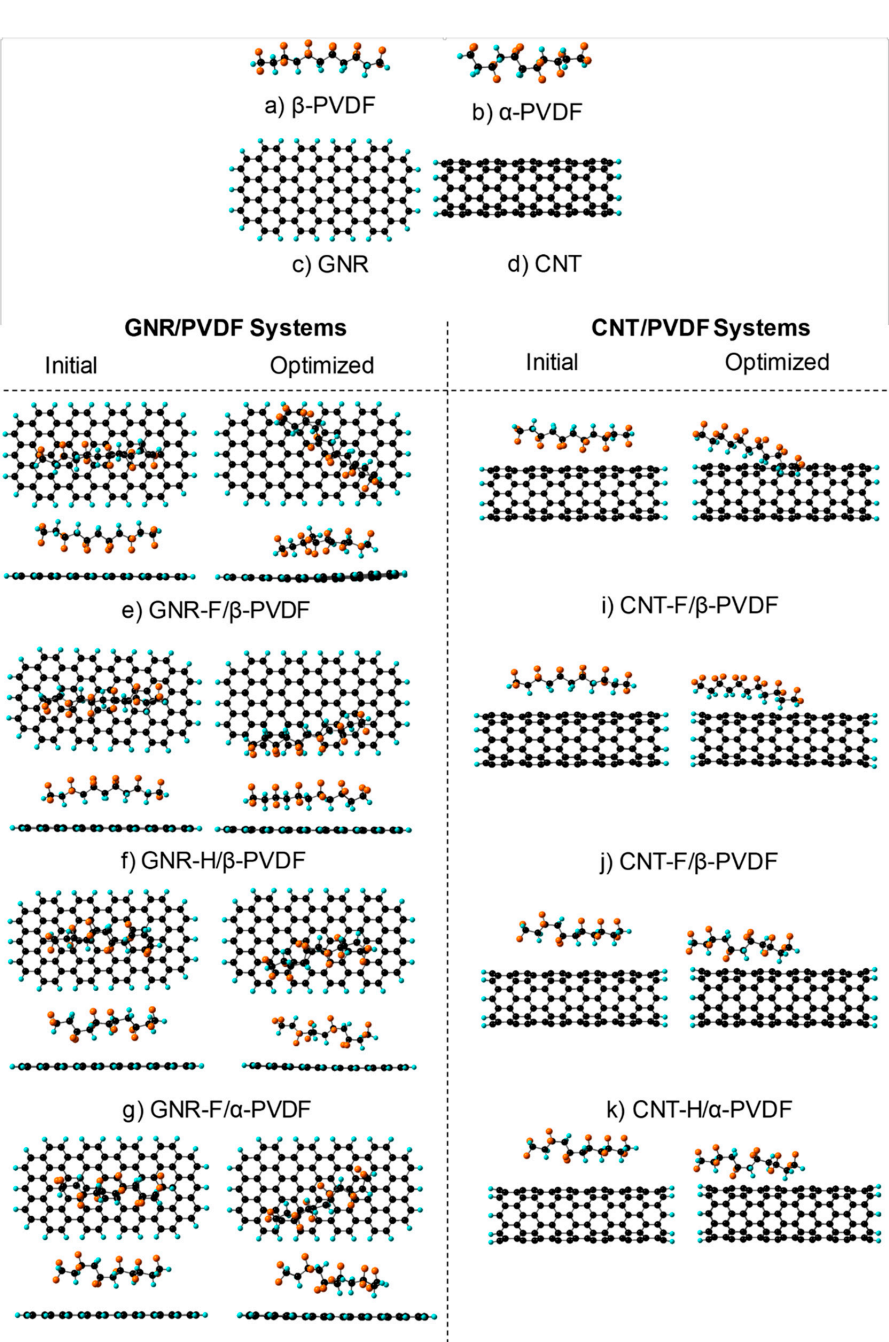
Optimized geometries of (**a**) β- polyvinylidene fluoride (PVDF), (**b**) α-PVDF, (**c**) graphene nanoribbon (GNR), and (**d**) carbon nanotube (CNT). Initial and optimized structures of (**e**–**h**) GNR/PVDF (left side) and (**i**–**l**) CNT/PVDF (right side). The atoms in black, blue and orange correspond to carbon, hydrogen, and fluorine, respectively.

The interactions between a segment of PVDF (both α and β configurations were considered) and the surface of a GNR and CNT were studied using molecular simulations. The GNR has an armchair configuration with a width and length of 10 Å and 20 Å, respectively. The simulated CNT is a zigzag (8,0) with a length of 20 Å. The dangling bonds of both nanomaterials were passivated with hydrogen atoms to avoid end effects. The segment of PVDF (head to head arrangement) is composed of six monomers, which were arranged to obtain α-PVDF or β-PVDF. The GNR, CNT and PVDF structures (Figure 1) were optimized to obtain equilibrium structures, which correspond to a global minimum in the potential surface energy (a frequency calculation was used to confirm the absence of imaginary frequencies).

The optimized PVDF segment was centered and localized at 4.0 Å from the surface of the optimized GNR or CNT. Two different configurations of the PVDF segment on the surface of the nanomaterials were considered: configuration H (nanomaterial-H/α- or β-PVDF), where the hydrogen atoms were directed towards the surface of the nanomaterials, and configuration F (nanomaterial-F/α- or β-PVDF), where the fluorine atoms were localized towards the surface of the nanomaterial. For α-PVDF, the segment was located towards the nanomaterial in a way that the maximum amount of hydrogen or fluorine atoms was facing the surface. The GNR/PVDF and CNT/PVDF systems were also optimized using a semi-empirical parameterized model 6 (PM6) [25] with the software Gaussian G09.

The strength of the interaction between the nanomaterials and the PVDF segments was evaluated by the concept of binding energy, which is defined as the difference between the summation of the energy of the constituents and the energy of the whole interacting system:

(1) ΔE=E(PVDF segment)+E(nanomaterial)−E(nanomaterial/PVDF)

where *E(PVDF segment)* and *E(nanomaterial)* are the energies of the optimized geometries of the PVDF, GNR and CNT, while *E(nanomaterial/PVDF)* corresponds to the energy of the GNR/PVDF and CNT/PVDF system. Based on the previous definition of the binding energy, an interaction is favorable if Δ*E* is positive.

### 2.3. Rheology

Rheological measurements were performed using an Anton–Paar MCR 302 rheometer (Montreal, Canada) at 240 °C using a 25 mm cone-plate geometry (a cone-tip angle of 1° and a truncation of 47 μm) under an inert N_2_ atmosphere. Test specimens were checked for thermal stability using oscillatory time-sweep experiments with long exposure times at the test temperature. 

### 2.4. Electrical Conductivity and EMI Shielding

The electrical conductivity of the rectangular samples was measured in the thickness direction. For the nanocomposites with an electrical conductivity higher than 10^−2^ S·m^−1^, the measurements were conducted according to the ASTM 257-75 standards using a Loresta GP resistivity meter (MCP-T610 model, Mitsubishi Chemical Co., Japan) connected with an ESP (four-pin) probe. In the four-pin probe technique, it is assumed that the sample is isotropic. Thus, the electrical conductivities in thickness and orthogonal directions are postulated to be the same. As the samples are compression molded, this assumption is rational. For electrical conductivities less than 10^−2^ S·m^−1^, the measurements were performed with a Keithley 6517A electrometer connected to a Keithley 8009 test fixture (Keithley Instruments, Beaverton, OR, USA). EMI shielding measurements in the X-band frequency range (8.2–12.4 GHz) were performed using an E5071C network analyzer (ENA series 300 KHz–20 GHz, Keysight Technologies, Santa Rosa, CA, USA). To perform the measurements, the samples (1.1 mm thickness) under the test were sandwiched between two waveguides of the network analyzer. The network analyzer sent a signal down the waveguide incident to the sample and then the scattering parameters (S-parameters) of each sample were recorded and used to measure EMI shielding effectiveness (EMI SE). EMI SE is defined as the logarithm of the ratio of incident power to transmitted power and is expressed in dB [26]. Since the power of each signal is proportional to the square of voltage magnitude, EMI SE is defined as the following:

(2) $$SE_{R}=10\times log\left(\frac{1}{1-R}\right)=10\times log\left(1/\left(1-{\left|S_{11}\right|}^{2}\right)\right)$$

(3) $$SE_{A}=10\times log\left(\frac{1-R}{T}\right)=10\times log\left(\frac{1-{\left|S_{11}\right|}^{2}}{{\left|S_{12}\right|}^{2}}\right)$$

(4) $$EMI\;SE=SE_{R}+SE_{A}$$

where *SE_R_*, *SE_A,_* and *EMI SE* are the shielding by reflection, shielding by absorption and overall shielding, respectively, and *R* and *T* are reflectance and transmittance, respectively. S_11_ and S_22_ are reflected over the incident voltage magnitude, whereas S_21_ and S_12_ signify transmitted over incident voltage magnitude in ports 1 and 2, respectively. The imaginary permittivity of the generated samples was also obtained by S-parameters conversion using Reflection/Transmission Mu and the Epsilon Nicolson-Ross Model.

## 3. Results and Discussion

### 3.1. Molecular Simulations

The simulation results in Table 1 show that α-PVDF has lower energy than β-PVDF; thus, α conformation is energetically more favorable than β. From the five well-known crystalline conformations of the PVDF, the α-PVDF is a more stable and abundant polymorph thermodynamically [27,28]. Comparison of GNR/PVDF and CNT/PVDF systems shows that both α-PVDF and β-PVDF have a stronger interaction (higher binding energy) towards CNT. These results were attributed to the curvature effect of CNT in a previous work [17]. In addition, it was found that the binding energy of all the nanomaterial/PVDF systems is higher for configuration H than for configuration F. This says that the interaction of both α-PVDF and β-PVDF is more favorable when the H atoms are directed towards the surface of the nanomaterials. In line with the current study, Zhao et al. [29] conducted AB-INITO simulations of hydrogen fluoride adsorption on graphene and claimed that the most stable configuration of HF on the graphene surface occurs when H points to the graphene surface. All in all, the molecular simulations results show that CNT would have a better interaction with the PVDF matrix in any configurations.

### 3.2. Rheological Response

#### 3.2.1. Linear Viscoelastic Behavior

Melt rheological measurements can uniquely provide in-depth information regarding the intricacies related to the flow response of polymeric systems. Percolated network structures formed by nanofillers introduce complex rheological behavior in polymer nanocomposites. Behavior such as a low-frequency solid-like behavior [30], thixotropy [31], and the emergence of prolonged relaxation processes [32] in the terminal region marks the traditional characteristics of these systems. Researchers long tried to attribute these hallmarks to a modified relaxational hierarchy related to polymer chains immobilized in the nanofiller/polymer interfacial region [33]. However, observation of a non-terminal rheology in weakly interacting systems has necessitated the importance of contact aggregation as a major contributor to the solid-like behavior in nanocomposites [34]. 

**Figure 2:**
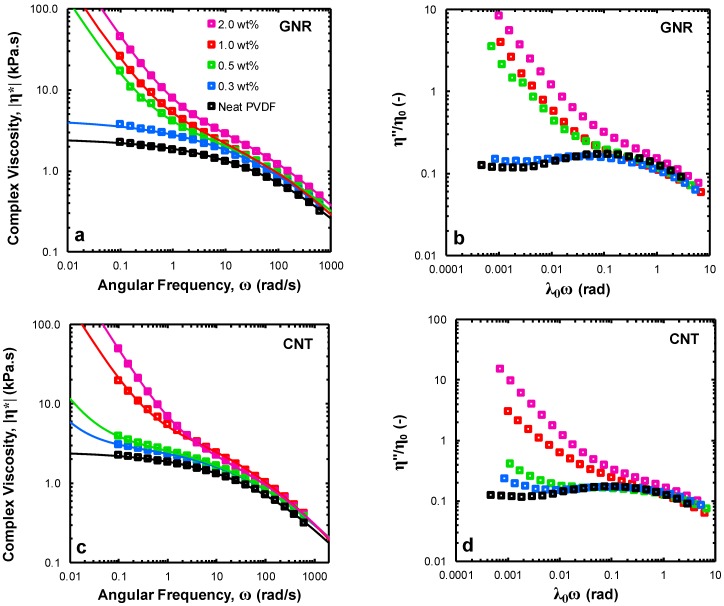
(**a**) Frequency dependence of the absolute magnitude of complex viscosity, (**b**) normalized out-of-phase component of complex viscosity $\frac{\eta^{\prime \prime }}{\eta_{0}}$ plotted as a function of reduced frequency $\lambda_{0}\omega$ for GNR/PVDF nanocomposites and (**c**) frequency dependence of the absolute magnitude of complex viscosity and (**d**) normalized out-of-phase component of complex viscosity $\frac{\eta^{\prime \prime }}{\eta_{0}}$ plotted as a function of reduced frequency $\lambda_{0}\omega$ for CNT/PVDF nanocomposites. All measurements were performed at a strain amplitude of $\gamma_{0}=$ 0.1% and T= 240 °C. Solid lines in (**a**,**c**) are best fits obtained using a modified 5-parameter Carreau model (see text for details).

Figure 2,c show the frequency dependence of the absolute magnitude of complex viscosity $\left|\eta^{*}\right|$ under a small amplitude oscillatory shear-field at a strain amplitude of $\gamma_{0}=$ 0.1% and T= 240 °C over a frequency range of 0.1 to 625 rad/s. As can be seen, in the low-frequency region, at higher concentrations for both GNR/PVDF and CNT/PVDF nanocomposites, the complex viscosity function tends to diverge to non-finite viscosity values and depart from the terminal (liquid-like) low-frequency response observable for the neat PVDF sample. To further depict the differences in the evolution of dynamic arrest and solid-like behavior in nanocomposite systems, complex viscosity data were fitted using a modified 5-parameter Carreau model as follows:

(5) $$\left|\eta^{*}\left(\omega \right)\right|=\frac{\eta_{0}}{{\left[1+{\left(\lambda_{0}\omega \right)}^{a}\right]}^{\frac{1-n}{a}}}+\frac{\tau_{y}}{\omega }$$

where $\eta_{0}$ is zero-shear viscosity, $\lambda_{0}$ is a characteristic relaxation time, the exponent a determines the breadth of transition from a Newtonian regime to a shear-thinning regime and n introduces the viscosity function slope at high frequencies. A yield-stress term, characterized by $\tau_{y},$ was added to this equation to consider the effect of non-terminal, low-frequency behavior in the nanocomposite systems [35]. The Carreau model parameters were utilized to reduce the axes and define a normalized representation based on the normalized out-of-phase component of complex viscosity $\frac{\eta^{\prime \prime }}{\eta_{0}}$ plotted as a function of reduced frequency $\lambda_{0}\omega$; this parameter emphasizes the evolution of a solid-like response in CNT/PVDF and GNR/PVDF nanocomposites. 

As can be seen in Figure 2b, the GNR/PVDF nanocomposites feature a sudden emergence of an upturn in $\frac{\eta^{\prime \prime }}{\eta_{0}}$ at low frequencies for near-percolation samples (i.e., 0.5 wt%). Contrarily, CNT/PVDF nanocomposites clearly exhibit a signature for the presence of a percolated nanofiller network (a diverging low-frequency $\frac{\eta^{\prime \prime }}{\eta_{0}}$), even at concentrations as low as 0.3 wt%, followed by a gradual intensification of the solid-like behavior. This is attributable to a smaller range of ribbon-ribbon short-range interactions compared to tube-tube short-range interactions, leading to stronger concentration-dependence for the rheological response of GNR/PVDF nanocomposites near the percolation threshold [36]. This proposes tube-tube mechanical entanglements as the main driver towards imparting the solid-like behavior in CNT/PVDF nanocomposites. A combination of polymer chain adsorption on the surface of nanofillers and chain confinement effect would be the dominant mechanisms underlying the low-frequency dynamic arrest in the case of GNR/PVDF nanocomposites. A detailed discussion regarding these mechanisms can be found elsewhere [16].

#### 3.2.2. Stress Relaxation Behavior

**Figure 3:**
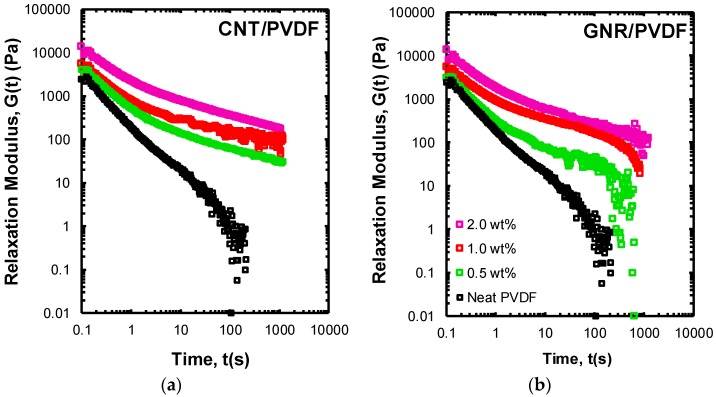
Stress dissipation upon application of a finite strain of γ = 10% for (**a**) CNT/PVDF and (**b**) GNR/PVDF nanocomposite samples at different concentrations at 240 °C. The stepper motor response time is 0.1 s.

To provide a more in-depth insight into the relaxational hierarchy and the origin of liquid-like to solid-like transition in the prepared nanocomposites, we performed the step-strain experiment displayed in Figure 3. In this experiment, a finite step-change in strain with a step-size of γ= 10% was applied and the pattern for stress relaxation was monitored in terms of the relaxation modulus G(t,γ) as a function of time. This strain level was selected in a way to be well within the linear viscoelastic region of the PVDF sample.

A neat PVDF melt showed a relaxation pattern consistent with a highly entangled polymer melt [37]. The relaxation mechanism for such a system is a complex combination of different mechanisms, including local Rouse dynamics, reptation-based relaxation modes, contour length fluctuation, and constraint release [12,13]. While Rouse dynamics are dominant for the short-time response, the long-time response of the neat PVDF is impacted by the presence of entanglements. Complete stress relaxation occurred for the neat PVDF in the first hundreds of seconds; however, the initial, intermediate, and long-time response of samples containing CNT and GNR nanomaterials clearly showed at least a prolonged or, in the case of CNT-based systems, an incomplete stress relaxation behavior. This can be attributed to the gel-like structures present in polymer nanocomposites as a colloidal suspension, where the recovery of percolated gelled structure and reformation of stress-bearing points and network junctions prevent complete stress relaxation. The GNR/PVDF nanocomposites clearly demonstrate a plateau at intermediate times, indicative of a slowed relaxation process compared to neat PVDF. This intermediate plateau is followed by complete relaxation and diminishing shear stress at longer exposure times. This can be suggestive of a deformation regime where network junctions are perturbed by local stresses and are no longer able to bear loading at longer times.

In contrast to GNR/PVDF samples, CNT/PVDF nanocomposite showed high resilience to the applied deformation-field, and the stress-bearing backbone of the fractal clusters survived over the entire time frame of the experiment. This resulted in the observation of an incomplete stress relaxation process for the CNT/PVDF nanocomposites. The result of this experiment confirms a fundamentally different set of mechanisms controlling short-range inter-particle interactions in the GNR/PVDF samples compared to CNT/PVDF samples. In fact, contrary to the CNT/PVDF system, the GNR network does not form through ribbon-ribbon direct contact and mechanical interlocking between neighboring GNRs [17,36]. Instead, primary and secondary entanglements between adsorbed polymer chains and bulk polymer chains is a more plausible assumption for dynamic arrest in the GNR/PVDF nanocomposites. In brief, the rheological results validate that CNTs feature a far stronger network (mechanical interlocking) than GNRs (primary and secondary entanglements between adsorbed polymer chains and bulk polymer chains).

### 3.3. Electrical Conductivity and EMI Shielding

In this section, we aim at assessing the effect of nanofiller geometry on electrical conductivity and EMI shielding of the generated nanocomposites. The electrical properties of CPNs depend on many factors such as content, inherent conductivity, size, and the aspect ratio of the conductive nanofiller, intrinsic properties of the polymer matrix, the quality of interaction between conductive nanofiller and polymer matrix, the dispersion state of nanofiller, mixing technique, and the crystallinity of the matrix, etc. [38,39,40,41]. In our previous study [17], we conducted optical microscopy, transmission electron microscopy (TEM) and differential scanning calorimetry on CNT/PVDF and GNR/PVDF nanocomposites, and observed comparable length and dispersion states in the nanofillers, and crystallinity of the polymer matrix. TEM also showed no signs of crimping, wrinkling, or rolling of GNRs within the PVDF matrix. In the same study, a powder conductivity measurement unveiled a fourfold higher electrical conductivity for GNR (40 S·m^−1^) compared to the parent CNT (10 S·m^−1^). Moreover, interior parent CNT walls are not accessible to interact with polymer chains and this gives rise to the chance of GNR to form a conductive network at lower nanofiller contents than CNTs. All the observations mentioned above suggest a lower electrical percolation threshold for GNR/PVDF than CNT/PVDF nanocomposites.

**Figure 4:**
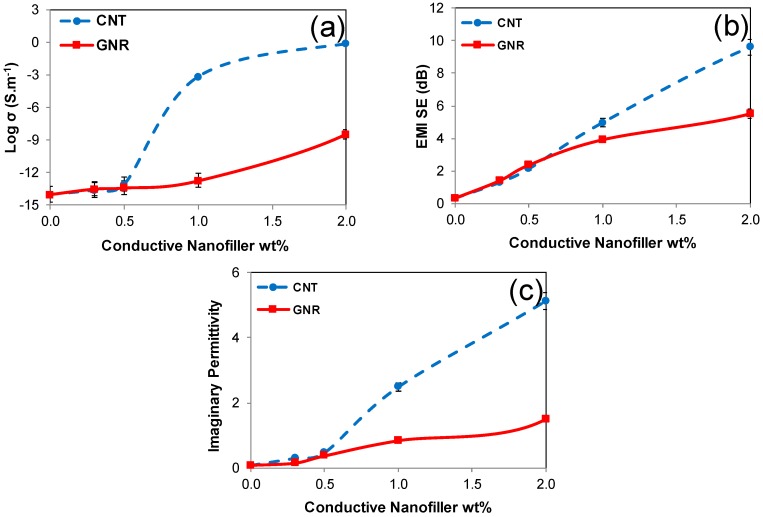
(**a**) Electrical conductivity, (**b**) electromagnetic interference (EMI SE) and (**c**) imaginary permittivity over the X-band (8.2–12.4 GHz) of parent CNT and GNR nanocomposites.

Contrary to forecast results based on the morphological and structural characterizations, Figure 4a denotes the significant superior electrical conductivity of the CNT/PVDF nanocomposites to the GNR/PVDF counterparts. These results are quite impressive due to the similar molecular structure but different geometries of CNT and GNR. As unearthed by rheology, we attribute this substantial discrepancy to the superior interlocking capability of parent CNT to GNR. It is worth noting that, as revealed by the molecular simulations, CNT has a better interaction with the PVDF matrix, which could also contribute to the formation of an enhanced conductive network.

Employing the percolation theory [42,43], we obtained an electrical percolation threshold of 0.9 wt% for the parent CNT/PVDF nanocomposites. A sharp upturn in electrical conductivity in the concentration range of 0.5–1.0 wt% is observable for the parent CNT nanocomposites, indicating the advent of conductive network formation. However, in the case of the GNR nanocomposites, the electrical conductivity increased gradually with GNR content, and thus the term pseudo-threshold behavior would be appropriate. Such behavior was already observed for GNP nanocomposites [12,44].

There are many types of contacts between GNR in a nanocomposite: plane to plane, edge to edge (cross or parallel), and edge to plane, among which plane-to-plane contact is the most helpful for electron transference [12]. Thus, as two-dimensional materials, GNRs are more difficult to interlock each other into a network structure than CNTs with a one-dimensional structure. In other words, GNRs approach each other and are in close contiguity, but are not able to interlace each other tightly, thereby leading to poorer electrical properties. This was well verified with the linear viscoelastic and stress relaxation behavior of the generated nanocomposites. 

Given their similar molecular structure but different geometries and network strengths, comparing the EMI shielding of CNT and GNR nanocomposites can benchmark the relationship between the nanofiller geometry and EMI shielding experimentally. EMI shielding in conductive nanocomposites consists of two primary mechanisms, viz., reflection and absorption. Reflection arises from the impedance mismatch between two media, i.e., two media with different electrical conductivities, real permittivities, and magnetic permeabilities. Absorption in conductive nanocomposites originates from Ohmic loss and polarization loss. Ohmic loss comes from the dissipation of energy by nomadic charges through conduction, hopping, and tunneling mechanisms, whereas polarization loss derives from the reorientation of electric dipoles in each half cycle of the electromagnetic wave [8].

Figure 4b shows that EMI shielding increased with nanofiller content for both types of nanocomposites but with unlike trends. EMI shielding increased with nanofiller content due to an enhanced number of dissipating mobile charge carriers in conjunction with an enhanced conductive network formation, i.e., an increase in reflection, Ohmic loss, and polarizations loss. More importantly, it can be seen that EMI SE of the parent CNT nanocomposites surpassed its GNR counterparts at 1.0 wt% and higher loadings. The concentration at which a conductive network is initiated to form a parent CNT is 1.0 wt%. These results confirm the direct relationship between the quality of the conductive network and EMI SE. Indeed, the superior ability of the parent CNT to interlock and thus generate an enhanced network compared to the GNR nanocomposites led to enhanced EMI shielding. It is worth mentioning that EMI SE at low filler contents, where there was no network formation, was slightly higher for GNR, which can be ascribed to higher powder conductivity of GNR. The energy dissipation by Ohmic loss is represented by the imaginary permittivity. Figure 4c clearly shows that the imaginary permittivity of CNT nanocomposites well surpassed that of the GNR nanocomposites at 1.0 wt%; this is in harmony with electrical conductivity and EMI shielding data. Given the higher power conductivity of GNR than CNT, imaginary permittivity data validate the role of nanofiller geometry on energy dissipation and EMI shielding.

**Figure 5:**
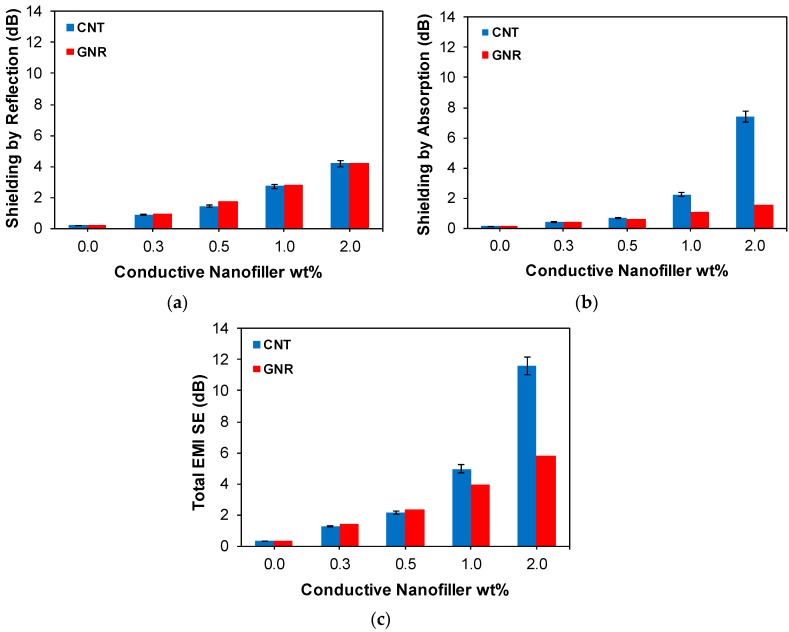
Comparison of (**a**) shielding by reflection, (**b**) shielding by absorption and (**c**) total EMI SE of CNT/PVDF and GNR/PVDF nanocomposites.

Shielding by reflection, shielding by absorption and total EMI SE of the CNT/PVDF and GNR/PVDF nanocomposites are presented in Figure 5. The results indicate that GNR/PVDF nanocomposites showcase a higher shielding by reflection than their CNT/PVDF counterparts at all nanofiller loadings. This can be related to higher powder conductivity and the larger surface area of GNR accessible to interact with an incident electromagnetic wave. It is worth noting that following the unzipping of multi-walled CNTs, the surface area of interior walls becomes available to interact with incident electromagnetic waves. Nevertheless, it can be observed that as the nanofiller loading increases, the effect of absorption gets dominant over reflection, and the CNT/PVDF nanocomposites outperform their GNR/PVDF counterparts. For instance, at 2.0 wt%, CNT/PVDF nanocomposites showed a shielding by absorption of 7.42 dB, which is over fourfold better than their GNR counterparts (1.68 dB). These results well verify the effect of the interlocking ability of CNTs and conductive network formation on shielding by absorption and total EMI SE.

## 4. Conclusions

For the first time in the field, this study compared the EMI shielding performance of CNT/PVDF and GNR/PVDF nanocomposites. Despite lower powder conductivity and comparable dispersion state, parent CNT/PVDF nanocomposites presented a lower electrical percolation threshold than GNR/PVDF nanocomposites. This was ascribed to an enhanced interlocking ability and thus stronger conductive network of CNTs, validated by molecular simulations and rheological assays. At low concentrations where there was no formed conductive network, GNR/PVDF nanocomposites presented higher EMI shielding, attributed to the higher powder conductivity and larger available interacting surface area of GNR. At higher concentrations where a conductive network was formed, CNT/PVDF nanocomposites showed a highly superior EMI shielding. Given the similar molecular structure but different geometries of CNT and GNR, higher EMI shielding of CNT/PVDF nanocomposites was attributed to the greater network formation ability of a tubular nanostructure than a ribbon-like nanostructure.

## Figures and Tables

**Table 1:** Summary of the energy, HOMO and LUMO energies, band gap, and binding energy of the studied systems.

System	Energy (Ha)	HOMO (eV)	LUMO (eV)	Band Gap (eV)	Binding Energy ∆*E* (Kcal/mol)	Imaginary Frequencies [*v*(cm^−1^)]
**β-PVDF**	−1.04568	−12.42	1.39	13.813	-	0
**α-PVDF**	−1.06076	−12.80	1.30	14.099	-	0
**GNR**	0.44717	−7.45	−2.21	5.241	-	0
**CNT**	2.20370	−5.60	−4.90	0.700	-	0
**GNR-F/β-PVDF**	−0.61176	−7.54	−2.31	5.226	8.32	0
**GNR-H/β-PVDF**	−0.61491	−7.82	−2.58	5.234	10.29	0
**CNT-F/β-PVDF**	1.06100	−6.43	−3.67	2.760	60.88	0
**CNT-H/β-PVDF**	1.03871	−7.11	−3.99	3.119	74.87	0
**GNR-F/α-PVDF**	−0.61883	−7.52	−2.30	5.224	3.29	0
**GNR-H/α-PVDF**	−0.62173	−7.58	−2.36	5.212	5.11	0
**CNT-F/α-PVDF**	1.03369	−6.68	−3.84	2.837	68.55	0
**CNT-H/α-PVDF**	1.03287	−6.79	−3.94	2.843	69.07	0

## Supplementary Materials

The following are available online at https://www.mdpi.com/2073-4360/11/6/1064/s1. Transmission electron microscopy of parent CNT and GNR; Representative schematics of fully and partially longitudinally unzipped CNT.

## References

[1] Thomassin J.-M., Jérôme C., Pardoen T., Bailly C., Huynen I., Detrembleur C. (2013). Polymer/carbon based composites as electromagnetic interference (emi) shielding materials. Mater. Sci. Eng. R Rep..

[2] Keteklahijani Y.Z., Arjmand M., Sundararaj U. (2017). Cobalt catalyst grown carbon nanotube/poly(vinylidene fluoride) nanocomposites: Effect of synthesis temperature on morphology, electrical conductivity and electromagnetic interference shielding. ChemistrySelect.

[3] Arjmand M., Moud A.A., Li Y., Sundararaj U. (2015). Outstanding electromagnetic interference shielding of silver nanowires: Comparison with carbon nanotubes. RSC Adv..

[4] Kolanowska A. (2018). From blackness to invisibility—Carbon nanotubes role in the attenuation of and shielding from radio waves for stealth technology. Carbon.

[5] Chukov D., Nematulloev S., Zadorozhnyy M., Tcherdyntsev V., Stepashkin A., Zherebtsov D. (2019). Structure, mechanical and thermal properties of polyphenylene sulfide and polysulfone impregnated carbon fiber composites. Polymers.

[6] Chukov D.I., Stepashkin A.A., Gorshenkov M.V., Tcherdyntsev V.V., Kaloshkin S.D. (2014). Surface modification of carbon fibers and its effect on the fiber–matrix interaction of uhmwpe based composites. J. Alloys Compd..

[7] TabkhPaz M., Mahmoodi M., Arjmand M., Sundararaj U., Chu J., Park S.S. (2015). Investigation of chaotic mixing for mwcnt/polymer composites. Macromol. Mater. Eng..

[8] Arjmand M., Chizari K., Krause B., Pötschke P., Sundararaj U. (2016). Effect of synthesis catalyst on structure of nitrogen-doped carbon nanotubes and electrical conductivity and electromagnetic interference shielding of their polymeric nanocomposites. Carbon.

[9] Xie S.H., Liu Y.Y., Li J.Y. (2008). Comparison of the effective conductivity between composites reinforced by graphene nanosheets and carbon nanotubes. Appl. Phys. Lett..

[10] Safdari M., Al-Haik M.S. (2013). Synergistic electrical and thermal transport properties of hybrid polymeric nanocomposites based on carbon nanotubes and graphite nanoplatelets. Carbon.

[11] Martin-Gallego M., Bernal M.M., Hernandez M., Verdejo R., Lopez-Manchado M.A. (2013). Comparison of filler percolation and mechanical properties in graphene and carbon nanotubes filled epoxy nanocomposites. Eur. Polym. J..

[12] Du J., Zhao L., Zeng Y., Zhang L., Li F., Liu P., Liu C. (2011). Comparison of electrical properties between multi-walled carbon nanotube and graphene nanosheet/high density polyethylene composites with a segregated network structure. Carbon.

[13] Genorio B., Lu W., Dimiev A.M., Zhu Y., Raji A.-R.O., Novosel B., Alemany L.B., Tour J.M. (2012). In situ intercalation replacement and selective functionalization of graphene nanoribbon stacks. ACS Nano.

[14] Jiao L., Zhang L., Wang X., Diankov G., Dai H. (2009). Narrow graphene nanoribbons from carbon nanotubes. Nature.

[15] Li X., Wang X., Zhang L., Lee S., Dai H. (2008). Chemically derived, ultrasmooth graphene nanoribbon semiconductors. Science.

[16] Arjmand M., Sadeghi S., Khajehpour M., Sundararaj U. (2017). Carbon nanotube/graphene nanoribbon/polyvinylidene fluoride hybrid nanocomposites: Rheological and dielectric properties. J. Phys. Chem. C.

[17] Sadeghi S., Arjmand M., Otero Navas I., Zehtab Yazdi A., Sundararaj U. (2017). Effect of nanofiller geometry on network formation in polymeric nanocomposites: Comparison of rheological and electrical properties of multiwalled carbon nanotube and graphene nanoribbon. Macromolecules.

[18] Rafiee M.A., Lu W., Thomas A.V., Zandiatashbar A., Rafiee J., Tour J.M., Koratkar N.A. (2010). Graphene nanoribbon composites. ACS Nano.

[19] Chung D.D.L. (2001). Electromagnetic interference shielding effectiveness of carbon materials. Carbon.

[20] Arjmand M., Apperley T., Okoniewski M., Sundararaj U. (2012). Comparative study of electromagnetic interference shielding properties of injection molded versus compression molded multi-walled carbon nanotube/polystyrene composites. Carbon.

[21] Ke K., Pötschke P., Jehnichen D., Fischer D., Voit B. (2014). Achieving β-phase poly(vinylidene fluoride) from melt cooling: Effect of surface functionalized carbon nanotubes. Polymer.

[22] Pawar S.P., Arjmand M., Gandi M., Bose S., Sundararaj U. (2016). Critical insights into understanding the effects of synthesis temperature and nitrogen doping towards charge storage capability and microwave shielding in nitrogen-doped carbon nanotube/polymer nanocomposites. RSC Adv..

[23] Eswaraiah V., Sankaranarayanan V., Ramaprabhu S. (2011). Functionalized graphene–pvdf foam composites for emi shielding. Macromol. Mater. Eng..

[24] Breuer O., Sundararaj U., Toogood R.W. (2004). The design and performance of a new miniature mixer for specialty polymer blends and nanocomposites. Polym. Eng. Sci..

[25] Stewart J.J.P. (2007). Optimization of parameters for semiempirical methods v: Modification of nddo approximations and application to 70 elements. J. Mol. Model..

[26] Mirkhani S.A., Arjmand M., Sadeghi S., Krause B., Pötschke P., Sundararaj U. (2017). Impact of synthesis temperature on morphology, rheology and electromagnetic interference shielding of cvd-grown carbon nanotube/polyvinylidene fluoride nanocomposites. Synth. Met..

[27] Güryel S., Walker M., Geerlings P., De Proft F., Wilson M.R. (2017). Molecular dynamics simulations of the structure and the morphology of graphene/polymer nanocomposites. Phys. Chem. Chem. Phys..

[28] Hasegawa R., Takahashi Y., Chatani Y., Tadokoro H. (1972). Crystal structures of three crystalline forms of poly(vinylidene fluoride). Polym. J..

[29] Zhao M., Lai Q., Xiao D., Guo Y. (2018). First-principles study on the adsorption of hf on reduced graphene oxide. ChemistrySelect.

[30] Trappe V., Sandkühler P. (2004). Colloidal gels—Low-density disordered solid-like states. Curr. Opin. Colloid Interface Sci..

[31] Vasu K.S., Krishnaswamy R., Sampath S., Sood A.K. (2013). Yield stress, thixotropy and shear banding in a dilute aqueous suspension of few layer graphene oxide platelets. Soft Matter.

[32] Sprakel J., Lindström S.B., Kodger T.E., Weitz D.A. (2011). Stress enhancement in the delayed yielding of colloidal gels. Phys. Rev. Lett..

[33] Lin C.-C., Gam S., Meth J.S., Clarke N., Winey K.I., Composto R.J. (2013). Do attractive polymer–nanoparticle interactions retard polymer diffusion in nanocomposites?. Macromolecules.

[34] Masser K.A., Yuan H., Karim A., Snyder C.R. (2013). Polymer chain dynamics in intercalated poly(ε-caprolactone)/nanoplatelet blends. Macromolecules.

[35] Filippone G., Carroccio S.C., Curcuruto G., Passaglia E., Gambarotti C., Dintcheva N.T. (2015). Time-resolved rheology as a tool to monitor the progress of polymer degradation in the melt state—Part ii: Thermal and thermo-oxidative degradation of polyamide 11/organo-clay nanocomposites. Polymer.

[36] Jiang T., Zukoski C.F. (2013). The effect of polymer-induced attraction on dynamical arrests of polymer composites with bimodal particle size distributions. J. Rheol..

[37] Venerus D.C., Nair R. (2005). Stress relaxation dynamics of an entangled polystyrene solution following step strain flow. J. Rheol..

[38] Bauhofer W., Kovacs J.Z. (2009). A review and analysis of electrical percolation in carbon nanotube polymer composites. Compos. Sci. Technol..

[39] Spitalsky Z., Tasis D., Papagelis K., Galiotis C. (2010). Carbon nanotube–polymer composites: Chemistry, processing, mechanical and electrical properties. Prog. Polym. Sci..

[40] Arjmand M., Sundararaj U. (2016). Impact of batio3 as insulative ferroelectric barrier on the broadband dielectric properties of mwcnt/pvdf nanocomposites. Polym. Compos..

[41] Hoseini A.H.A., Arjmand M., Sundararaj U., Trifkovic M. (2017). Significance of interfacial interaction and agglomerates on electrical properties of polymer-carbon nanotube nanocomposites. Mater. Des..

[42] Weber M., Kamal M.R. (1997). Estimation of the volume resistivity of electrically conductive composites. Polym. Compos..

[43] Abbasi S., Carreau P.J., Derdouri A. (2010). Flow induced orientation of multiwalled carbon nanotubes in polycarbonate nanocomposites: Rheology, conductivity and mechanical properties. Polymer.

[44] Liang J., Wang Y., Huang Y., Ma Y., Liu Z., Cai J., Zhang C., Gao H., Chen Y. (2009). Electromagnetic interference shielding of graphene/epoxy composites. Carbon.

