# Supplementary material for: Carbon Nanotube versus Graphene Nanoribbon: Impact of Nanofiller Geometry on Electromagnetic Interference Shielding of Polyvinylidene Fluoride Nanocomposites
Source: Polymers (Basel). 2019 Jun 20;11(6):1064. doi: 10.3390/polym11061064 (PMC6632034; doi:10.3390/polym11061064)
Supplement: Supplementary file 1 [file polymers-11-01064-s001.pdf]

Supporting Information for

Carbon Nanotube versus Graphene Nanoribbon: Impact of Nanofiller Geometry on Electromagnetic Interference Shielding of Polyvinylidene Fluoride Nanocomposites

Mohammad Arjmand[a,ⱡ*], Soheil Sadeghi[b,ⱡ], Ivonne Otero Navas[b], Yalda Zamani Keteklahijani[b], Sara Dordanihaghighi[a], Uttandaraman Sundararaj[b*]

[a]School of Engineering, University of British Columbia, Kelowna, BC, V1V 1V7 Canada

[b]Department of Chemical and Petroleum Engineering, University of Calgary, Calgary, AB, T2N 1N4, Canada

*Corresponding authors. Mohammad Arjmand (mohammad.arjmand@ubc.ca); Uttandaraman Sundararaj (u.sundararaj@ucalgary.ca)

ⱡMA and SS made equal contribution to this work.

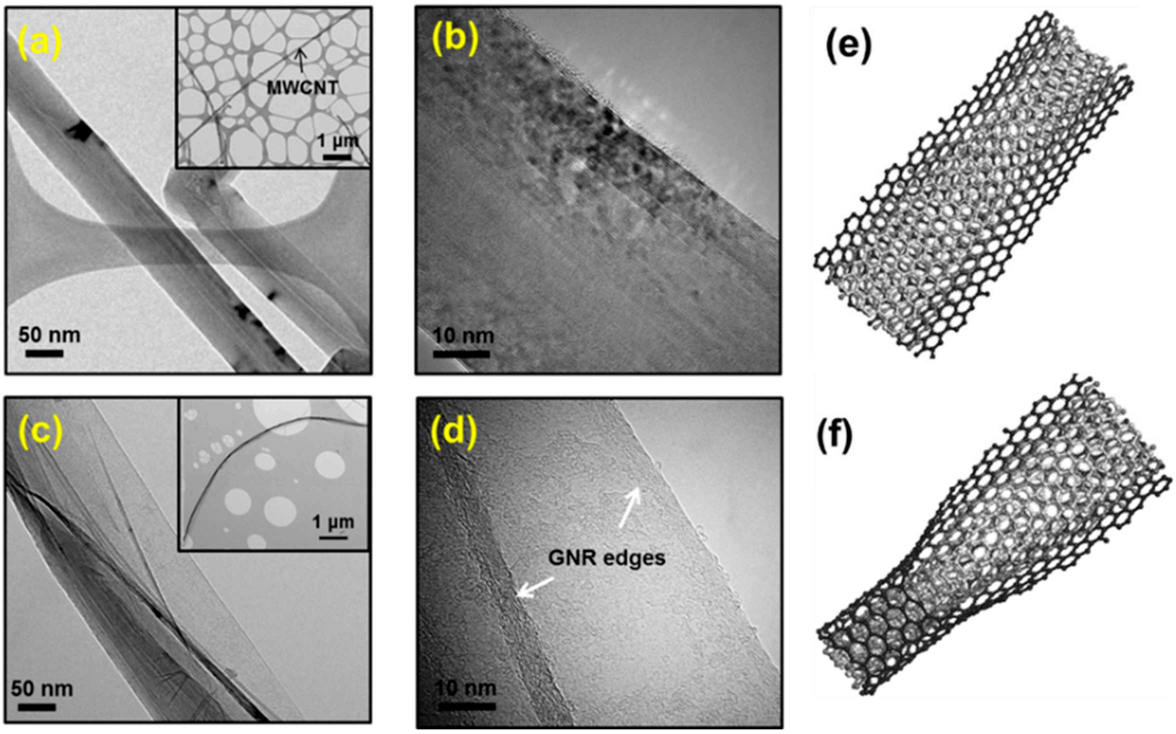

Figure S1. (a) Low-magnification and (b) high-magnification TEM image of parent CNT. (c) Low-magnification and (d) high-magnification TEM image of GNR. (e) and (f) show schemes of fully and partially longitudinally unzipped CNT, respectively. Adapted with permission from Sadeghi et al. Macromolecules, 2017; 50: 3954-3967 [1]. Copyright (2017), American Chemical Society.

References

1. Sadeghi, S.; Arjmand, M.; Otero Navas, I.; Zehtab Yazdi, A.; Sundararaj, U., Effect of Nanofiller Geometry on Network Formation in Polymeric Nanocomposites: Comparison of Rheological and Electrical Properties of Multiwalled Carbon Nanotube and Graphene Nanoribbon. *Macromolecules* **2017**, *50*, 3954-3967.
